# Assessing the effect of mandatory progress reporting on treatment requirements identified during health examinations at the Fukushima Daiichi Nuclear Power Plant: A time series analysis

**DOI:** 10.1002/1348-9585.12111

**Published:** 2020-01-22

**Authors:** Koji Mori, Seiichiro Tateishi, Tatsuhiko Kubo, Yuichi Kobayashi, Ko Hiraoka, Futoshi Kawashita, Takeshi Hayashi, Yoshifumi Kiyomoto, Masaki Kobashi, Kota Fukai, Hiroyuki Tahara, Ryuji Okazaki, Akira Ogami, Kazuyuki Igari, Katsunori Suzuki, Hiroshi Kikuchi, Kazuhiro Sakai, Toru Yoshikawa, Yoshihisa Fujino

**Affiliations:** ^1^ Department of Occupational Health Practice and Management Institute of Industrial Ecological Sciences University of Occupational and Environmental Health Kitakyushu Japan; ^2^ Health Center University of Occupational and Environmental Health Kitakyushu Japan; ^3^ Department of Environmental Epidemiology Institute of Industrial Ecological Sciences University of Occupational and Environmental Health Kitakyushu Japan; ^4^ Occupational Health Training Center University of Occupational and Environmental Health Kitakyushu Japan; ^5^ Hitachi Health Care Center Hitachi, Ltd. Hitachi Japan; ^6^ Personnel Department Kumagai Gumi Co., Ltd. Tokyo Japan; ^7^ Department of Preventive Medicine School of Medicine Tokai University Isehara Japan; ^8^ Department of Mental Health Institute of Industrial Ecological Sciences University of Occupational and Environmental Health Kitakyushu Japan; ^9^ Department of Radiological Health Science Institute of Industrial Ecological Sciences University of Occupational and Environmental Health Kitakyushu Japan; ^10^ Department of Work Systems and Health Institute of Industrial Ecological Sciences University of Occupational and Environmental Health Kitakyushu Japan; ^11^ Igari Occupational Health Consultant Office Tokyo Japan; ^12^ Division of Infection Control and Prevention University of Occupational and Environmental Health Hospital Kitakyushu Japan; ^13^ Health Administration Center Business Solution Company Tokyo Electric Power Company Holdings Tokyo Japan; ^14^ The Ohara Memorial Institute for Science of Labour Kawasaki Japan; ^15^ National Institute of Occupational Safety and Health Kawasaki Japan

**Keywords:** decommissioning, fitness for duty, health examination, nuclear accident, occupational health

## Abstract

**Objectives:**

At the Fukushima Daiichi Nuclear Power Plant, run by the Tokyo Electric Power Company, new procedures were introduced as part of the fitness for duty program in July 2016. These were designed to ensure that treatment and further investigations identified as necessary during health examinations were carried out. This study aimed to assess the effectiveness of the initiative by testing whether workers who needed further health examinations obtained them promptly, and whether the number with unmet health needs decreased and the number of workers being treated increased.

**Methods:**

The primary contractors reported aggregated quarterly results of health examinations of both their own and their subcontractors’ employees, and follow‐up visits to medical institutions were also reported over the next two quarters. The study used data for the period from July 2016 to December 2018. Incident rate ratios were estimated using a multilevel Poisson regression model, including the logarithm of the number of workers who took health examination for each primary contractor company as offset. The linear trend was assessed by treating the number of periods as a continuous variable.

**Results:**

The incident rate ratio for workers who needed treatment having a follow‐up examination promptly showed a significant decrease over time. The incident rate ratio for those with unmet needs decreased, and those being treated increased over time.

**Conclusions:**

The findings showed that the initiative was effective, with the number of early visits for further health examinations increasing and a decrease in the number of people with unmet health needs.

## 1 INTRODUCTION

A nuclear accident classified as Level 7 (serious accident) on the International Nuclear Event Scale occurred at the Fukushima Daiichi Nuclear Power Plant run by the Tokyo Electric Power Company (TEPCO) as a result of the Tohoku Region Pacific Coast Earthquake of March 11, 2011 and the resulting tsunami. Immediately after the accident, a significant number of workers employed by TEPCO, nuclear reactor manufacturers, general construction companies, and subcontractors were engaged in reactor stabilization, decontamination, and decommissioning operations. Preventive measures against radiation hazards, heatstroke, and pandemics were put in place and strengthened. Many workers were working under complex command and control structures such as multiple subcontracts, but the measures were effective in preventing the development of significant health issues.^1^, ^2^ Many medical institutions around the power plant were closed following the disaster, and the journey time to secondary and tertiary emergency hospitals was considerable, so a 24‐hour emergency medical office was established in the plant.^3^ However, there have been a number of incidents of workers dying at work from myocardial infarction and stroke, suggesting that improved measures were needed to check fitness for duty and provide preventive advice.^1^, ^4^

Enhancement of the fitness for duty procedures was resisted by the companies concerned because of the complexity and the possible impact on recruitment and retention.^4^ From October 2012, however, new workers were required to have a doctor's certificate that they were “permitted to work at the nuclear power plant,” based on the results of a health examination. Primary contractors were responsible for this procedure among subcontractors. We proposed that the procedure was extended to all workers operating for a particular length of time, with periodic health examinations, but this was not implemented because it would place the primary contractors under a responsibility beyond the legal requirements.^4^ Despite these procedures, however, there were three fatal cases of sudden cardiac arrest on the site of the power plant between August and September 2015. The Fukushima Labor Bureau considered the situation seriously, and gave TEPCO and the primary contractors an administrative instruction to ensure the health of workers. TEPCO responded that it would examine and implement countermeasures with guidance from the University of Occupational and Environmental Health, Japan (UOEH). UOEH advised TEPCO that five conditions were necessary to ensure the health of workers:

1. All workers needed to undergo a regular health examination;
2. All workers who were assessed as needing treatment or further examination should visit a medical institution;
3. All workers who needed treatment should continue to receive that treatment at least while they were working at the nuclear power plant;
4. If judged necessary followed a periodic health examination, work restrictions, or changes to accommodate a health condition would be put in place for individuals; and
5. The work restrictions or accommodations undertaken would be periodically reviewed and revised.

In April 2016, TEPCO asked all primary contractors to establish a system to confirm the achievement of the five conditions within their organization, and to operate the system from July that year. Contractors reported to TEPCO on the new procedures at the end of November, in a summary report on health examinations for the third quarter (July to September) of 2016.^4^ The report included the numbers judged to “need further examination,” “need treatment,” and “need ongoing treatment,” and the numbers visiting a doctor for further examination and treatment on a quarterly basis.

Outcome indicators were needed to evaluate the effects of this initiative. One option was to use the number of cases of myocardial infarction and cerebrovascular disorder as a direct outcome. However, the numbers were considered too small to see trends and it is also difficult to collect information on events that occur away from the workplace. There was also no basis for collecting data such as blood pressure, or blood sugar levels. We therefore examined two hypotheses to test the effects:

1. Workers who are judged to require further examination will visit more promptly than before; and
2. The number of workers receiving treatment will increase, and the number who need treatment but do not receive it (that is, have unmet health needs) will decrease.

## 2 METHODS

### 2.1 Target companies and workers

Workers at the nuclear power plant can be divided into employees of TEPCO, employees of primary contractor companies, contracted directly with TEPCO, and employees of their subcontractors, possibly over several layers of outsourcing. The target companies in this study were the primary contractors and their subcontractors, in the period from July 2016 to December 2018. The only exceptions were two companies that operated staff cafeterias and convenience stores. The target workers were all those employed in the target companies who underwent a general health examination during the period. Most of them were registered as radiation workers and were subject to both regular (6‐monthly) health examinations.

### 2.2 Procedures for checking the results of health examinations

Each primary contractor and subcontractor either used the local medical institutions selected collectively by the primary contractors, or chose their own to provide health examinations for their employees. The judgment for each employee was made by the doctor who carried out their health examination. The judgment criteria were not unified, but categories had to include “need further examination,” “need treatment,” and “need ongoing treatment.” In some companies, occupational health physicians reviewed and reassessed the results.

Tokyo Electric Power Company required the primary contractors to report quarterly aggregated results of their own and their subcontractors’ health examinations. These reports had to be submitted within two months of the end of the period. For example, the deadline for reporting on the second quarter of the year (from April to June) was the end of August. Reporting items included the number of heath examinations received, the numbers of workers who needed further examination, treatment or ongoing treatment and whether the further examination had been completed at the time of the report. The primary contractors were also asked to report whether the further examination had been completed and the status of those judged to “need treatment” in the next two quarterly reports. If there were many cases that had not yet had a further examination or treatment, TEPCO suggested that the primary contractor should improve their system.

### 2.3 Data analysis

This is a time series analysis. The total values for the primary contractor and all its subcontractors were used in each analysis. For the hypothesis that workers who need further examinations will visit more promptly, we counted anyone who had not yet received this further examination at the initial reporting point. For the hypothesis about increasing numbers of workers being treated, and decreasing numbers with unmet needs, we assessed the numbers judged as “need treatment” and “need ongoing treatment.” Most of the workers underwent a health examination every 6 months, so semiannual recipients are considered to be highly homogenous populations. For the analysis of “need treatment” and “need ongoing treatment,” we therefore used semi‐annual data instead of quarterly data, summing figures for the first quarter (Q1) and second quarter (Q2) for the first half of the year (H1), and the third and fourth quarter (Q3 and Q4) for the second half of the year (H2).

Incident rate ratios (IRR) for each hypothesis were estimated using a multilevel Poisson regression model, including the logarithm of the number of workers who took health examination for each primary contractor company as offset because effects were at the company level. The linear trend was assessed by treating the number of periods as a continuous variable in the Poisson regression model. We used STATA15 for statistical analysis.

### 2.4 Ethical considerations

The activities were conducted based on general medical norms, and this research used the records of these activities. The researchers did not handle any personal information.

## 3 RESULTS

The number of eligible primary contractors was 47 in the third quarter (July to September) of 2016, and it increased to 55 in the firth quarter (October to December) of 2018. All target companies reported necessary data on the relevant period and did at least two time points, but some of them had periods when there were no workers who had a health examination. However, although the number of workers undergoing health examinations varied across quarters during the study period, it tended to decrease overall. The proportion of those who were judged to “need further examination” varied from 9.6% in the second quarter of 2017 to 4.7% in the third quarter of 2018. The proportion of those who needed treatment and ongoing treatment ranged from 3.3% to 2.1% and 16.9% to 9.7%. The number who were assessed as needing further examinations who had not undergone those examinations by the reporting deadline two months after the end of the quarter (the number of “further examinations not completed”) varied from 6 to 191 people. The ratio of those needing a further examination also varied, from 4.7% to 9.6% (Table 1; See Appendix for basic statistics).

**Table 1:** Results of general health examinations

Period	No. of primary contractors		No. of total health exam	Need further exam		Need treatment		Need ongoing treatment		Further examination not completed	
	Total	With health exam data		n	%^a^	n	%^b^	n	％^c^	N	％^d^
2016 Q3	47	46	4859	282	5.8	161	3.3	720	14.8	57	20.2
2016 Q4	51	42	6421	605	9.4	185	2.9	623	9.7	191	31.6
2017 Q1	51	48	4834	266	5.5	121	2.5	687	14.2	33	12.4
2017 Q2	51	49	6376	609	9.6	135	2.1	755	11.8	169	27.8
2017 Q3	50	50	4104	264	6.4	137	3.3	694	16.9	28	10.6
2017 Q4	51	50	6043	504	8.3	124	2.1	849	14.0	93	18.5
2018 Q1	51	49	3848	244	6.3	116	3.0	580	15.1	15	6.1
2018 Q2	52	50	5444	497	9.1	114	2.1	723	13.3	87	17.5
2018 Q3	55	54	3458	164	4.7	83	2.4	500	14.5	6	3.7
2018 Q4	55	52	5221	440	8.4	113	2.2	736	14.1	64	14.5

a Those needing further examination as a percentage of total numbers undergoing health examinations.

b Those needing treatment as a percentage of total numbers undergoing health examinations.

c Those needing ongoing treatment as a percentage of total numbers undergoing health examinations.

d Those not completed a further examination as a percentage of those needing one.

The IRR for those needing an examination who had not yet had it by the end of the reporting period showed a significant decrease between the third quarter of 2016 and any quarters in 2017 and 2018 except for the second quarter of 2017. The same trend was seen across the entire study period (IRR 0.95, 95% confidence interval [CI] 0.92‐0.98; *P* < .001) (Table 2). The IRR for the numbers needing treatment showed a significant decrease in every half year and that for those needing ongoing treatment a significant increase in every half year except for the first half of 2017. Across the entire study period, the IRR was 0.91 (95% CI 0.87‐0.95; *P* < .001) for needing treatment and 1.06 (95% CI 1.04‐1.07; *P* < .001) for needing ongoing treatment, showing significant trends (Table 3).

**Table 2:** Time trend for those needing a further examination who had not received one

	Further examination not completed			
	IRR^a^	95% CI		*P* value
2016 Q3	Reference			
2016 Q4	0.81	0.60	1.10	.175
2017 Q1	0.60	0.39	0.93	.021
2017 Q2	0.75	0.55	1.03	.072
2017 Q3	0.57	0.36	0.90	.015
2017 Q4	0.64	0.46	0.90	.009
2018 Q1	0.51	0.29	0.90	.021
2018 Q2	0.69	0.49	0.98	.037
2018 Q3	0.26	0.11	0.61	.002
2018 Q4	0.58	0.40	0.83	.003
Trend	0.95	0.92	0.98	<.001

Abbreviation: CI, confidence interval.

a Incident rate ratio.

**Table 3:** Time trend for judgments of whether workers needed treatment and ongoing treatment

	Need treatment				Need ongoing treatment			
	IRR^a^	95% CI		*P* value	IRR^a^	95% CI		*P* value
2016 H2	reference				reference			
2017 H1	0.65	0.55	0.76	<.001	1.05	0.97	1.13	.229
2017 H2	0.71	0.60	0.83	<.001	1.31	1.22	1.41	<.001
2018 H1	0.69	0.59	0.82	<.001	1.20	1.12	1.30	<.001
2018 H2	0.63	0.53	0.75	<.001	1.22	1.13	1.32	<.001
Trend	0.91	0.87	0.95	<.001	1.06	1.04	1.07	<.001

Abbreviation: CI, confidence interval.

a Incident rate ratio.

## 4 DISCUSSION

At the nuclear power plant, we carried out work to confirm whether treatment and further examinations identified as necessary during workers’ health examinations were carried out promptly. We found that the reporting and checking procedures had the effect of ensuring that those who were considered to need further examinations were more likely to have received those investigations. The new procedures were also associated with increasing numbers being treated, and decreasing numbers with unmet needs.

Since the nuclear accident occurred, various efforts have been made to prevent adverse effects on workers’ health.^1^ However, improving the fitness for duty procedures was resisted because of the perceived burden and concerns about the impact on staffing. In the wake of three onsite sudden deaths in the nuclear power plant, the Fukushima Labor Bureau issued TEPCO with an administrative instruction on improving health management measures. In response, TEPCO developed a system of thorough checks on the results of health examinations, including whether further investigations and treatment were required, and whether these had happened, with the technical support of occupational health specialists at UOEH.^4^ The operation of the system required significant work from many stakeholders, so it was considered important to assess its effects on workers’ health. It is not easy to use outcome indicators such as reduction in myocardial infarctions or cerebrovascular disease, so the evaluation used two alternative indicators.

First, we looked at whether any further examinations had been completed by the reporting deadline each quarter, two months after the end of the quarter. For those who had not completed these further examinations, a progress report was required in the next two quarters. With the exception of the workers who left the plant during the period, all workers received any further examination during that period. If this initiative continues, we expect to see improved awareness among representatives and workers in both primary contractors and subcontractors, so that workers needing further examinations receive them more quickly. A positive effect was observed in this study. Previous studies have reported that guidance on consultation and follow‐up procedures for health examinations affected behavior associated with further examinations.^5^, ^6^ A significantly larger number of employees in small‐to‐medium‐sized workplaces visited medical institutions for further examinations after receiving instructions to do so from public health nurses.^5^ More employees also visited medical institutions when a public health center issued a letter of introduction after an adult medical examination.^6^

Second, we assumed that if the same workers have continued in post, and those needing treatment for chronic diseases have started to receive care, then the number of workers receiving treatment would increase. This effect was observed in this study. A previous study found that medical management is improved by occupational health intervention: continuation of treatment for hypertension and the control of hypertension and diabetes mellitus were better in workplaces with full‐time occupational health staff such as occupational health physicians and occupational health nurses than in a workplace without full‐time occupational health staff.^7^ This study did not examine information from the health examination or the effect of treatment on disease control. It did not consider what items of health examinations required treatment or further examination, either. However, it is expected that better continuation of treatment will also improve the control of chronic conditions.

The five guidance items that were the basis of this initiative were designed to deliver appropriate management of medical matters and fitness for duty assessment. This could therefore be regarded as a high‐risk approach to disease prevention. Unfortunately, several sudden onsite deaths have occurred even since the initiative has been running. In those cases, the results of the health examination and follow‐up decisions were immediately reviewed, confirming that appropriate actions were taken based on the procedures. The high‐risk approach alone has limitations in preventing the occurrence of these events,^8^ so it may be necessary to consider health guidance for those at lower risk, education to improve the health literacy of workers, and other population approaches.

This study had several limitations. There were some differences between the procedures used in different companies, such as the criteria for health judgments, and information obtained from subcontractors. In some primary contractors, company occupational health physicians reevaluated the results of the health examinations. So, existence of occupational physician might be a confounding factor. However, we did not have the information. Since assignment of an occupational physician is required based on worksite size rather than company size, the number of workers who took health examination was considered in the model as a proxy of worksite size. During the observation period, there were also some changes in the target population, such as new starters or employees leaving one of the companies, and some new primary contractors. These changes were due to a decrease in construction work in the nuclear plant and other business reasons. A decrease in the number of employees may have caused a healthy work effect which may have strengthened the results of the efforts, and an increase in the number of primary contractors may have weakened the results. This was an observational study, and therefore the trends observed cannot be clearly identified as the effects of the initiative. Measures addressing factors in the work environment and work load are also considered important in preventing sudden death in decommissioning work in nuclear power plants, but this study did not consider those factors.

However, despite these limitations, this study has provided useful findings on healthcare support to reduce the risk of sudden death among workers engaged in difficult tasks under a complex employment structure. There have been a few previous studies on occupational health support for workers providing post‐disaster responses.^9^ This study observed that changes in medical management approaches can also be effective, as well as the health risk measures previously reported,^1^, ^2^ and therefore adds valuable information about countermeasures in nuclear power plants and in response to future disasters.

## 5 CONCLUSION

Introducing reporting procedures about action on the healthcare requirements identified during health examinations had a positive effect on outcomes such as faster uptake of further examinations and fewer unmet health needs among workers in a nuclear power plant undergoing decommissioning. This initiative should therefore be continued. However, the approach used limits the effectiveness of disease prevention, and further health management efforts including population approaches are needed.

## DISCLOSURE

*Approval of the research protocol:* N/A. *Informed consent:* N/A. *Registry and registration no. of the study/trial:* N/A. *Animal studies:* N/A. *Conflicts of interest*: HK is an employee of TEPCO. KI has a contract with TEPCO. TH and MK are employees of one of the primary contractors. FK and KI have contracts with one of the primary contractors.

## AUTHOR CONTRIBUTIONS

KM and ST conceptualized and designed the study; KM, ST, TK, Y.Kobayashi, KH, FK, TH, Y.Kiyomoto, MK, KF, HT, RO, AO, KI, K.Suzuki, HK, K.Sakai, and TY contributed to the development of advice and recommendations to TEPCO during the period; KM and YF analyzed and interpreted the data; KM led the writing; ST, TK, Y.Kobayashi, KH, FK, TH, Y.Kiyomoto, MK, KF, HT, RO, AO, KI, K.Suzuki, HK, K.Sakai, TY, and YF critically reviewed the manuscript.
