# Supplementary material for: Assessing the effect of mandatory progress reporting on treatment requirements identified during health examinations at the Fukushima Daiichi Nuclear Power Plant: A time series analysis
Source: J Occup Health. 2020 Jan 22;62(1):e12111. doi: 10.1002/1348-9585.12111 (PMC6976892; doi:10.1002/1348-9585.12111)
Supplement: Supplementary file 1 [file JOH2-62-e12111-s001.docx]

Appendix 1. Basic statistics on numbers of total health examinations

| period | No. of Primary contractors | Mean | SD | Minimum | 25%tile | 50%tile | 75%tile | maximum |
| --- | --- | --- | --- | --- | --- | --- | --- | --- |
| 16_Q3 | 46 | 106 | 158 | 5 | 18 | 43.5 | 105 | 667 |
| 16_Q4 | 42 | 153 | 249 | 2 | 20 | 56.5 | 185 | 1158 |
| 17_Q1 | 48 | 101 | 153 | 1 | 17.5 | 34.5 | 97.5 | 623 |
| 17_Q2 | 49 | 130 | 215 | 1 | 14 | 41 | 127 | 1115 |
| 17_Q3 | 50 | 82 | 130 | 1 | 12 | 32 | 81 | 723 |
| 17_Q4 | 50 | 121 | 196 | 1 | 14 | 29.5 | 137 | 1021 |
| 18_Q1 | 49 | 79 | 120 | 1 | 13 | 33 | 66 | 533 |
| 18_Q2 | 50 | 109 | 197 | 1 | 13 | 28.5 | 112 | 1190 |
| 18_Q3 | 54 | 64 | 106 | 1 | 10 | 32 | 70 | 563 |
| 18_Q4 | 52 | 100 | 176 | 1 | 12 | 29.5 | 109.5 | 1077 |

| Appendix 2, Basic statistics on numbers of needing further examinations | | | | | | | | | |
| --- | --- | --- | --- | --- | --- | --- | --- | --- | --- |
| period | No. of Primary contractors | Mean | SD | Minimum | 25%tile | 50%tile | 75%tile | maximum |  |
| 16_Q3 | 46 | 6 | 12 | 0 | 0 | 2 | 6 | 55 |  |
| 16_Q4 | 42 | 14 | 34 | 0 | 0 | 2 | 8 | 157 |  |
| 17_Q1 | 48 | 6 | 12 | 0 | 0 | 1 | 5 | 59 |  |
| 17_Q2 | 49 | 12 | 31 | 0 | 0 | 2 | 6 | 164 |  |
| 17_Q3 | 50 | 5 | 11 | 0 | 0 | 1.5 | 5 | 63 |  |
| 17_Q4 | 50 | 10 | 20 | 0 | 0 | 1.5 | 10 | 88 |  |
| 18_Q1 | 49 | 5 | 14 | 0 | 0 | 1 | 5 | 95 |  |
| 18_Q2 | 50 | 10 | 24 | 0 | 0 | 2 | 7 | 146 |  |
| 18_Q3 | 54 | 3 | 7 | 0 | 0 | 1 | 3 | 45 |  |
| 18_Q4 | 52 | 8 | 19 | 0 | 0 | 1 | 5.5 | 98 |  |

| Appendix 3. Basic statistics on numbers of needing treatment | | | | | | | | | |
| --- | --- | --- | --- | --- | --- | --- | --- | --- | --- |
| period | No. of Primary contractors | Mean | SD | Minimum | 25%tile | 50%tile | 75%tile | maximum |  |
| 16_Q3 | 46 | 4 | 8 | 0 | 0 | 1 | 3 | 46 |  |
| 16_Q4 | 42 | 4 | 11 | 0 | 0 | 1.5 | 4 | 67 |  |
| 17_Q1 | 48 | 3 | 6 | 0 | 0 | 1 | 2.5 | 42 |  |
| 17_Q2 | 49 | 3 | 7 | 0 | 0 | 1 | 3 | 49 |  |
| 17_Q3 | 50 | 3 | 7 | 0 | 0 | 0 | 2 | 50 |  |
| 17_Q4 | 50 | 2 | 5 | 0 | 0 | 0 | 2 | 28 |  |
| 18_Q1 | 49 | 2 | 4 | 0 | 0 | 1 | 3 | 19 |  |
| 18_Q2 | 50 | 2 | 4 | 0 | 0 | 0 | 2 | 23 |  |
| 18_Q3 | 54 | 2 | 2 | 0 | 0 | 0 | 2 | 10 |  |
| 18_Q4 | 52 | 2 | 5 | 0 | 0 | 0 | 2.5 | 35 |  |

| Appendix 4. Basic statistics on numbers of needing ongoing treatment | | | | | | | | | |
| --- | --- | --- | --- | --- | --- | --- | --- | --- | --- |
| period | No. of Primary contractors | Mean | SD | Minimum | 25%tile | 50%tile | 75%tile | maximum |  |
| 16_Q3 | 46 | 16 | 31 | 0 | 1 | 5 | 15 | 161 |  |
| 16_Q4 | 42 | 15 | 29 | 0 | 1 | 4.5 | 14 | 168 |  |
| 17_Q1 | 48 | 14 | 34 | 0 | 2 | 5 | 12.5 | 227 |  |
| 17_Q2 | 49 | 15 | 26 | 0 | 2 | 5 | 20 | 145 |  |
| 17_Q3 | 50 | 14 | 24 | 0 | 1 | 5.5 | 15 | 119 |  |
| 17_Q4 | 50 | 17 | 28 | 0 | 1 | 4.5 | 21 | 128 |  |
| 18_Q1 | 49 | 12 | 22 | 0 | 0 | 4 | 14 | 140 |  |
| 18_Q2 | 50 | 14 | 24 | 0 | 2 | 3.5 | 18 | 116 |  |
| 18_Q3 | 54 | 9 | 19 | 0 | 0 | 3 | 9 | 128 |  |
| 18_Q4 | 52 | 14 | 25 | 0 | 1 | 4 | 14.5 | 126 |  |

| Appendix 5 Basic statistics on numbers of workers who had not completed a further examination | | | | | | | | | |
| --- | --- | --- | --- | --- | --- | --- | --- | --- | --- |
| period | No. of Primary contractors | Mean | SD | Minimum | 25%tile | 50%tile | 75%tile | maximum |  |
| 16_Q3 | 46 | 1 | 5 | 0 | 0 | 0 | 0 | 29 |  |
| 16_Q4 | 42 | 5 | 19 | 0 | 0 | 0 | 0 | 91 |  |
| 17_Q1 | 48 | 1 | 3 | 0 | 0 | 0 | 0 | 22 |  |
| 17_Q2 | 49 | 3 | 16 | 0 | 0 | 0 | 0 | 93 |  |
| 17_Q3 | 50 | 1 | 2 | 0 | 0 | 0 | 0 | 17 |  |
| 17_Q4 | 50 | 2 | 9 | 0 | 0 | 0 | 0 | 48 |  |
| 18_Q1 | 49 | 0 | 1 | 0 | 0 | 0 | 0 | 10 |  |
| 18_Q2 | 50 | 2 | 8 | 0 | 0 | 0 | 0 | 51 |  |
| 18_Q3 | 54 | 0 | 1 | 0 | 0 | 0 | 0 | 3 |  |
| 18_Q4 | 52 | 1 | 6 | 0 | 0 | 0 | 0 | 33 |  |
